# Dengue disease surveillance: an updated systematic literature review

**DOI:** 10.1111/tmi.12333

**Published:** 2014-05-28

**Authors:** S Runge-Ranzinger, P J McCall, A Kroeger, O Horstick

**Affiliations:** 1 Special Programme for Research and Training in Tropical Diseases, World Health OrganizationGeneva, Switzerland; 2 Liverpool School of Tropical MedicineLiverpool, UK; 3 Institute of Public Health, University of HeidelbergHeidelberg, Germany

**Keywords:** dengue, surveillance, systematic review, epidemic preparedness, dengue outbreaks

## Abstract

**Objectives:**

To review the evidence for the application of tools for dengue outbreak prediction/detection and trend monitoring in passive and active disease surveillance systems in order to develop recommendations for endemic countries and identify important research needs.

**Methods:**

This systematic literature review followed the protocol of a review from 2008, extending the systematic search from January 2007 to February 2013 on PubMed, EMBASE, CDSR, WHOLIS and Lilacs. Data reporting followed the PRISMA statement. The eligibility criteria comprised (i) population at risk of dengue, (ii) dengue disease surveillance, (iii) outcome of surveillance described and (iv) empirical data evaluated. The analysis classified studies based on the purpose of the surveillance programme. The main limitation of the review was expected publication bias.

**Results:**

A total of 1116 papers were identified of which 36 articles were included in the review. Four cohort-based prospective studies calculated expansion factors demonstrating remarkable levels of underreporting in the surveillance systems. Several studies demonstrated that enhancement methods such as laboratory support, sentinel-based reporting and staff motivation contributed to improvements in dengue reporting. Additional improvements for passive surveillance systems are possible by incorporating simple data forms/entry/electronic-based reporting; defining clear system objectives; performing data analysis at the lowest possible level (e.g. district); seeking regular data feedback. Six studies showed that serotype changes were positively correlated with the number of reported cases or with dengue incidence, with lag times of up to 6 months. Three studies found that data on internet searches and event-based surveillance correlated well with the epidemic curve derived from surveillance data.

**Conclusions:**

Passive surveillance providing the baseline for outbreak alert should be strengthened and appropriate threshold levels for outbreak alerts investigated. Additional enhancement tools such as syndromic surveillance, laboratory support and motivation strategies can be added. Appropriate alert signals need to be identified and integrated into a risk assessment tool. Shifts in dengue serotypes/genotype or electronic event-based surveillance have also considerable potential as indicator in dengue surveillance. Further research on evidence-based response strategies and cost-effectiveness is needed.

**Objectifs:**

Analyser les résultats de l'application d'outils pour la prédiction/détection des épidémies de dengue et la surveillance des tendances dans les systèmes de surveillance active et passive des maladies, afin d’élaborer des recommandations pour les pays endémiques et identifier les besoins importants de recherche.

## Introduction

Dengue remains a major and growing public health threat worldwide. With the most recent study estimating that global infection rates of 390 million infections occur annually (Bhatt *et al.* 2013), the need for improved dengue surveillance is evident. Dengue surveillance is essential for the detection of outbreaks and, in the longer term, to monitor disease trends. In order to trigger timely interventions, outbreak alerts are particularly important to mobilise vector control and to prime or reorganise healthcare delivery services in preparation for a surge in suspected cases. Although vector control tools can be effective in principle, implementation remains an issue and effective dengue routine prevention is rarely achieved, particularly in high-density urban communities (Horstick *et al.* 2010; Pilger *et al.* 2010). Emergency vector control operations in response to dengue outbreaks are more typically applied, even though the efficacy of the most widespread method used, insecticide fogging or space-spraying, is dubious (Esu *et al.* 2010). Surveillance for dengue can include different indicators and systems (Harrington *et al.* 2013; Henning 2004; Stroup *et al.* 1989) to detect outbreaks and monitor trends. The authors' earlier systematic literature review (Runge Ranzinger *et al.* 2008) analysed ‘the evidence on the structure, purpose and usefulness of dengue disease surveillance in dengue endemic countries’ and described a general lack of evidence for the usefulness of dengue disease surveillance for early outbreak detection, especially the lack of indicators/alert signals available to trigger response. A stepwise adaptation of surveillance systems after evaluation in combination with active surveillance was recommended. Since then, new articles in the field of dengue surveillance have been published, and new initiatives towards early detection of dengue outbreaks have been launched (WHO expert meeting on dengue outbreak detection and response, June 2012). Common to all reports and recommendations is an increased recognition of the need for dengue control to focus on surveillance, vector control and adequate case management.

The aims of this review were to strengthen the evidence base and (where required) adjust the recommendations of Runge Ranzinger *et al*. (2008) by incorporating new trends and tools and to identify and summarise advances or improvements made. As in the earlier review, dengue vector surveillance was not covered here, but has been reviewed independently elsewhere (Bowman *et al.* 2014, in press).

## Methodology

This review followed the protocol (York 2001) used in a previous systematic literature review (Runge Ranzinger *et al.* 2008) on dengue disease surveillance, and the reporting guidelines set out in the PRISMA Statement for systematic reviews and meta-analyses (Liberati *et al.* 2009). The eligibility criteria of the reviewed literature were as follows: (i) population at risk of dengue, (ii) dengue disease surveillance, (iii) outcome of surveillance described and (iv) empirical data evaluated. After the recent dengue outbreaks in France and Croatia (29 and 30), the population at risk in the search was extended to include the European region. Literature reported in English, German and Spanish was included although the search was conducted in English only. Studies focusing on risk mapping, transmission dynamics, forecasting or prediction methods were excluded (e.g. Buczak *et al.* 2012; Chen & Chang 2013; Racloz *et al.* 2012), as they did not directly study surveillance systems.

The literature search and analyses were developed and continued until 15th February 2013, with two persons working as data extractors. Search fields included Medical Subject Heading (MeSH) terms/subjects and free text, considering population, intervention and outcome. The terms ‘dengue’ and ‘severe dengue’ (dengue fever (DF), dengue h(a)emorrhagic fever (DHF) and dengue shock syndrome (DSS)), ‘surveillance’ (disease, clinical, active, passive, sentinel, epidemiologic, population), ‘communicable disease control’, ‘effectiveness’, ‘evaluation’, ‘disease notification’, ‘disease outbreaks’, ‘hospital and clinical laboratory information system’ were used. The search strategy was adapted according to the databases, consistent with the process undertaken during the primary review published in 2008.

The search strategy was applied to the following databases to locate peer-reviewed studies: The United States National Library of Medicine and the National Institutes of Health Medical Database (PubMed) (1966–2013), Excerpta Medica Database (EMBASE) (1983–2013), the Cochrane Database of Systematic Reviews (CDSR), the World Health Organization (WHO) library database (WHOLIS) and the Latin American and Caribbean Health Sciences Database (Lilacs) (1967–2013). The references cited by relevant literature, including grey literature, were also screened for further articles. Grey literature and unpublished studies were included if found relevant to the research question and if they fulfilled the inclusion and exclusion criteria.

All results were screened for duplication by author, title, journal and publication date, and then screened for relevance, based on the title and abstract only. The full text of all studies considered to be relevant was then reviewed for final assessment by two independent data extractors. Where necessary, consensus was achieved by discussion. Relevant information, including study bibliographic information, study design and objectives, levels of endemicity and population, components of the surveillance system (surveillance subjects, scope and method), resources spent on the system, delivery of the surveillance system (information flow, outbreak and/or case definition, additional relevant information), purpose of the surveillance system and outcome attributes, was extracted and tabulated in evidence tables (Table1).

**Table:** Evidence tables

Author Publication year, Study population	Purpose & type (active/passive) of surveillance	Study design & objectives	Development & delivery of surveillance system	Outbreak definition, Case definition	Results and outcome attributes	Conclusions of study authors and risk of bias
*A. Surveillance for outbreak prediction and/or early outbreak detection*						
1. Chan EH et al. (2011) Bolivia, Brazil, India, Indonesia and Singapore (2003–2010)	To complement traditional surveillance by potentially facilitating earlier detection, capturing health-seeking behaviour, as well as capturing the population of the ill who do not seek medical care formally.	To build models able to estimate a disease activity indicator using data on Google search patterns fit to a time series of case counts from official data sources.	Aggregating historical anonymised logs of online Google search queries submitted between 2003 and 2010. Time series are computed for the most common search queries in the selected countries, irrespective of query language. Each time series was normalised by dividing the count for each query on a particular day by the total number of online search queries submitted.	Spikes in the time series indicate an increase in interest in dengue. To determine whether they are ‘true spikes’ or ‘spurious spikes’ (e.g. panic driven) can be distinguished when the rate of growth exceeds the normal rate of spread as determined by the basic reproduction number R0 or if p was found to exceed five standard deviations from the mean.	Model-fitted ‘expected’ epidemic curves generally matched ‘observed’ epidemic curves quite well for all five countries, with the exception of Bolivia in 2007 when the model overestimated the activity in that season, and India in 2005 for which it underestimated *Timeliness:* Potential to provide earlier signals of epidemics without delay of official case counts *Sensitivity:* Underreporting (e.g. due to misdiagnosis or subclinical cases) extends to the models as well; however, it could be a source of information for those otherwise not demanding health care at all or in the reporting sector.	**Conclusions of study author** - Could supplement traditional surveillance. - Would be a low-cost option - Estimating an indicator near real-time - Dengue-related search queries are generally not as influenced by news coverage (panic-driven searches) - Despite strong historical correlations, it remains susceptible to false alerts. - Sufficient search volume is needed. - Rural areas and developing nations tend to lack or have limited Internet access currently. - Intercountry comparisons may be difficult, each presented country and model must be considered independently.
2. Althouse BM et al. (2011) Singapore (weekly incidence, 2004–2011) and Bangkok (monthly incidence, 2004–2011)	Google search query	Dengue incidence data and Internet search data for the same period were downloaded. Search terms were chosen. Three models to predict incidence were compared.	Logistic regression and support vector machine (SVM) models were used to predict a binary outcome. Incidence prediction models were assessed using r2 and Pearson correlation. Logistic regression and SVM model performance were assessed. Models were validated using multiple cross-validation techniques.	NA	- In Bangkok, the model has an r2 ˜0:943, and a correlation of 0.869 between fitted and observed. - In Singapore, the model has an r2 ˜0:948, and a correlation of 0.931. - In both Singapore and Bangkok, SVM models outperformed logistic regression in predicting periods of high incidence. - The AUC for the SVM models using the 75th percentile cut-off is 0.906 in Singapore and 0.960 in Bangkok. - Our predictions of time periods with high dengue incidence are very accurate with sensitivities and specificities of 0.861–1.00 and 0.765–1.00 for multiple thresholds in each location.	**Conclusions of study author** - Internet search terms predict incidence of dengue with high accuracy. - The methods use freely available data and analysis tools and can be readily adapted to other settings**.** - In settings, with less developed surveillance systems, an internet search term-based system may yield significant gains in the rapidity of predictions. - It is conceivable that an internet search term-based model may be a proxy for routine surveillance in specific settings. - Individual models need to be developed for specific settings using local surveillance data and search terms.
3. Lee KS et al. (2010) Singapore 2006–2008	2005 laboratory-based dengue virus surveillance was established for close monitoring and investigation of the circulating dengue virus serotypes.	To proof a serotype switch for the 2007/2008 outbreak.	Phylogenetic analysis of DENV sequences was conducted using the maximum-likelihood method as implemented and compared with sequence data obtained from GenBank.	Warning level = 256 cases/epidemiologic week as reported by the Ministry of Health.	- The numbers of dengue-positive samples serotyped were 186 in 2006, 889 in 2007 and 918 in 2008 and represent ≈10% of the total dengue cases - During January–September 2006, 75%–100% of samples collected each month contained DENV-1. - In early January 2007, the predominant circulating serotype switched from DENV-1 to DENV-2. - The proportion of DENV-2-positive samples detected by PCR rose from 57.9% in January 2007 to a peak of 91.0% in July 2007. - Early detection of this switch warned of a possible upcoming dengue outbreak.	**Conclusions of study author** - Dengue surveillance provided early warning and contributed to early activation of enhanced vector control. - Unable to assess the effectiveness of the control measures, considering the regional situation in 2007, without these measures the dengue situation in Singapore in 2007 would have been worse than or comparable to that in 2004–2005. - Demonstrates how rapidly dengue virus serotypes can be replaced within a population.
4. Lee KS et al. (2012) Singapore January 2008–December 2010	A laboratory-based dengue virus surveillance programme established since 2005 provides an opportunity to study the circulating dengue viruses in this island state.	This study aims to understand the dynamics of dengue viruses in cosmopolitan Singapore. Envelope protein gene sequences of all four dengue serotypes (DENV-1–DENV-4) obtained from human sera in Singapore (2008–2010) were performed and analysed.	Clinical blood samples were collected from hospitals and general practitioner (GP) clinics from dengue-suspected patients. Real-time PCR (RT-PCR) for dengue RNA detection and serotyping was carried out in Environmental Health Institute (EHI) according to its in-house protocol.	PCR positive for DENV	- Of a total of 6515 samples (2008 –2010) from general practitioners and hospitals, 994 samples were positive for dengue by RT-PCR. All 4 serotypes were detected with DENV-2 (80.5%) continuing as the predominant serotype. DENV-1 (8.7%) and DENV-3 (8.2%) were also regularly detected while DENV-4 was rare (2.4%). - 380 (38.2%) samples were sequenced and analysed, the 255 DENV 2 Egene sequences obtained revealed that all but 22 were closely related to clade II of the cosmopolitan genotype that were associated with the 2007 dengue outbreak this clade II lineage has further expanded since 2007, into two separate newer clades; designated here as clades III and IV. Notably, by 2010, the two newer clades (III and IV) replaced clade II to be the predominant virus and were involved in the larger clusters occurring in Singapore, particularly at the end of 2010.	**Conclusions of study author:** - At the point of writing (July 2011), Singapore was experiencing a significant rise in number of cases, which were predominantly due to clade III and IV of cosmopolitan DENV-2. Reaching about 260 cases per week, it represented the highest number of cases per week since the 2007 outbreak and was equivalent to or more than twice the weekly numbers documented in the same period in 2008–2010. Suggesting that current data show that a replacement of a predominant viral clade, even in the absence of a switch in predominant serotype, could signal a possible increase in dengue transmission.
5. Koh Benjamin KW et al. (2008) Singapore 2005	Population-wide routine reporting including laboratory components.	Epidemiological, entomological and virological data were analysed retrospectively.	All medical practitioners should notify all cases and death of dengue to the MOH within 24 h by fax or via a website. Laboratories are also required to notify MOH of all patients whose blood samples tested positive for acute dengue infection.	A cluster is defined as 2 or more cases epidemiologically linked by place of residence or work/ school (within 150 m) and time (onset of illness within 14 days).	- A total of 1190 clusters involving 5362 epidemiologically linked cases were identified. This constituted 38.3% of all reported cases. The mean number of cases in each cluster was 3 (range, 2 to 75), and the mean duration of transmission was 5 days (range, 1 to 54). - A significant correlation between weekly mean temperature and cases was noted. The correlation was strong when the increase in temperature preceded rise in cases by a period of 18 weeks (*r *=* *0.60; *P* < 0.001)	**Conclusions of study author:** Factors contributing to this resurgence included - lower herd immunity and - the predominant dengue serotype for 2001–2003 was DEN-2. This was replaced by DEN-1 in June 2004 although this strain had been circulating in Singapore since 2002. This change in dengue serotype could have exposed a significant proportion of the population who may be immunologically naive to the new circulating serotype, although this is difficult to prove conclusively. - There was no evidence from gene sequencing of the dengue viruses that the epidemic was precipitated by the introduction of a new virulent strain.
6. Schreiber MJ et al. (2009) Singapore April–November 2005	Population-wide routine reporting including laboratory components.	By exploiting genomic data from an intensively studied major outbreak, molecular epidemiology of DENV at a fine-scaled temporal and spatial resolution is analysed.	All medical practitioners should notify all cases and death of dengue to the MOH within 24 h by fax or via a website. Laboratories are also required to notify MOH of all patients whose blood samples tested positive for acute dengue infection (from study 5).	A cluster is defined as 2 or more cases epidemiologically linked by place of residence or work/school (within 150 m) and time (onset of illness within 14 days) (from study 5).	133 RT-PCR dengue-positive patients collected; 66 (48.9%) DENV-1, 62 (46.6%) DENV-3 and 5 DENV-2 (3.8%). All but one of the DENV-1 genomes from this epidemic were classified as genotype I. The majority of DENV-3 genomes fell into genotype III, and an isolate from genotype III was first detected in Singapore in 2003 which fell basal to the 2005 outbreak viruses in our phylogenetic analysis.	- Epidemic surveillance of viral genome sequences in this case would not have been sufficient to predict the 2005 outbreak. - Concurrent surveillance of viral isolates, mosquito vectors and periodic surveys of seroprevalence rates of the population may therefore provide the additional required predictive information. - The chance discovery of the DENV-3 outbreak also highlights the value of comprehensive city-wide fever surveys in detecting rare events.
7. Yamanaka A (2011) Indonesia Surabaya 2007–2010	Virus surveillance, study based	Three surveys in Surabaya during: (i) April 2007, (ii) June 2008 to April 2009 and (iii) September 2009 to December 2010. A total of 231 isolates from dengue patients examined by PCR typing. Phylogenetic analyses were performed randomly.	Samples from 1071 patients aged from four months to 14 years, who were clinically diagnosed with DF or DHF. The association between DENV type and disease severity was evaluated by the chi-square test with the Yates’ correction factor. The probability value of *P* (0.05) was considered statistically significant.	Positive PCR for DENV	- We found that the predominant DENV shifted from type 2 to type 1 between October and November 2008. - All 22 selected isolates in the second survey belonged to genotype IV, and all 17 selected isolates in the third survey belonged to genotype I, indicating a genotype shift between April and September 2009. - The proportion of DHF cases increased about three times after the type shift in 2008. - The subsequent genotype shift in 2009 was associated with the increased number of total dengue cases.	**Conclusions of study author:** - This study shows a quick type shift of the predominant circulating DENV from DENV2 to DENV1 in Surabaya between October and November 2008. - 10% of dengue cases were linked with DHF in 2008, increasing to 28% in 2009. - We also found a DENV1 genotype shift from IV to I between April 2009 and September 2009, less than one year after displacement of the viral type - The total number of dengue patients increased in 2010, with 2169, 2268 and 3379 cases in 2008, 2009 and 2010, respectively.
8. Li DS et al. (2010) 2007–2009 Pacific Region	Not stated	During 1997–2000, the serotype was almost exclusively DENV-2, but during 2000–2001, <1 year, DENV-2 was displaced by multiple genotypes of DENV-1. Rapid replacement of DENV-1 by DENV-4 during 2008 is described.	Routine reporting discovered increased transmission with DENV-4 introduction: May 2008 in Kiribati outbreak, July 2008 Samoa, December 2008 shift from DENV-1 to DENV-4 in Tonga, November 2008 DENV-4 in New Caledonia, February 2009 French Polynesia. Phylogenetic analysis was performed	Not stated	- The chronology of the recovery of DENV-4 from patients in the region suggests that DENV-4 was introduced from Indonesia/Malaysia before 2007. - The relative genetic homogeneity of the DENV-4 recovered during this most recent outbreak in the Pacific region suggests introduction of a single genotype rather than introduction of multiple genotypes and to different locations, as was the case with DENV-1.	**Conclusions of study author:** - Outbreaks are initiated by the introduction of DENV, but the populations of most island nation states are too small to sustain transmission of a single DENV serotype for >4–5 years. - Interisland mobility in this region ensures rapid spread of any newly introduced viruses. - This synchronisation of spread may reflect the relatively small populations of most island states (≈250 000 residents), high attack rates and a high birth rate (≈30% of the population is <14 years) - If only 1 DENV serotype circulates at any time, and serotype replacement occurs approximately every 5 years, these data suggest that ≈30% (75 000) of 250 000 susceptible hosts are sufficient in these settings to support a serotype replacement and that DENV-3 may reappear in the Pacific island states in ≈2012. >
9. Rocha Claudio et al. 2009 Iquitos, Peru May 2000–August 2003	Two active surveillance systems, study based as cohorts, a) monitoring school absenteeism among the students b) community-based programme of door-to-door febrile surveillance in study neighbourhoods.	To better understand the epidemiology of dengue transmission in Iquitos, multiple active surveillance systems to detect symptomatic infections were established.	a)1100 children were recruited into a school-based febrile surveillance programme and monitored in school daily, during vacation periods once per week. b)5000 neighbourhood residents were invited to participate in a door-to-door febrile illness surveillance programme. Health workers interviewed each residence three times	If a child was absent from school, a home visit was made to determine whether the absence was because of febrile illness (≥ 38 °C).	- Febrile episodes were detected by both systems with equal rapidity after disease onset. - During the period that both programmes were running simultaneously in 2004, a higher number of febrile cases in general (4.52/100 versus 1.64/100 person-years) and dengue cases specifically (2.35/100 versus 1.29/100 person-years) were detected in school-aged children through the community-based surveillance programme. Similar results were obtained by direct comparison of 435 participants concurrently enrolled in both programmes (*P* < 0.005).	**Conclusions of study author:** The community-based programme captured twofold more fever and symptomatic dengue infections relative to study population size than the school-based system while monitoring five times as many people using the same number of personnel and the same amount of resources. - Community-based surveillance allowed to identify symptomatic dengue cases in all age groups and was not solely limited to school-aged children. - Several factors, including the research objective, site-specific dengue epidemiology and cultural characteristics of the study population, will help determine the type of active surveillance system to implement.
10. Meynard Jean-Baptiste et al. (2008) French Guiana week 41 of 2005 to week 25 of 2006.	Syndromic and clinical surveillance reporting on the armed forces and laboratory surveillance reporting on the civilian population.	The objectives were to study the value of a syndromic surveillance system set-up within the armed forces, compared with the traditional clinical surveillance system during this outbreak. The main studied performance was the early warning capacity.	a)Until 2006, surveillance was based on the weekly civilian laboratory surveillance of confirmed cases within the 200 000 general population. b)For the armed forces, the surveillance is based on the clinical military mandatory system within the 3000 soldiers. c)To enhance this clinical surveillance, a new syndromic prototype was set up in October 2004 combined with the clinical system.	a)Threshold= 6 cases per week for more than 2 consecutive weeks. b)A weekly statistical non-automated analysis using the Current Past Experienced Graph (CPEG) to compare with historical data. c)Data analysis is automated and uses both the CPEG and the Exponential Weighted Moving Average.	- Syndromic surveillance detected the dengue fever outbreak several weeks before clinical surveillance, a pre-alarm was activated during week 41, this was not confirmed for three more weeks. The real alarm with the armed forces started during week 44 of 2005. - On the civilian side, more time was necessary to detect the new dengue fever outbreak, in particular because it did not use any statistical tool to identify an increase in cases above a threshold. - Several weeks were also necessary for the local vector borne disease committee to request a strengthening of existing vector control measures.	**Conclusions of study author:** - Laboratory-based and syndromic surveillance is complementary; both contribute different surveillance data and together allow a better assessment of the epidemiological situation. - The addition of syndromic surveillance required the involvement of numerous new contributors. - It allowed an estimation of the impact of the 2006 outbreak, recording 16 200 suspected cases, whereas the previous system counted only 2500 confirmed cases. - However, syndromic surveillance is associated with an increased risk of false alarms and of system saturation in case of outbreak.
11. Jefferson Henry (2008) French Guiana Jan 2005–Dec 2006 Armed Forces	Syndromic fever surveillance (2SE FAG) system has been in operation since October 2004 and is a prototype, near real-time syndromic surveillance system operating among some 3000 armed forces in French Guiana.	The aim of this study was to evaluate the syndromic system using the CDC guidelines ‘Framework for Evaluating Public Health Surveillance Systems for Early Detection of Outbreaks’.	The system is designed to allow, in near real-time, geolocation and epidemiological analysis of cases of fever (temperature, >38 °C). Interviews within two main stakeholder groups of data input and data analysis personnel have been performed. A quantitative part investigated validity of reporting.	Suspected dengue: sudden onset of fever with no evidence of other infection (particularly malaria), associated with one or more non-specific symptoms including headache, myalgia, arthralgia and/or retro-orbital pain.	**Timeliness**: Ideal within 60 min. Delays and non-reporting due to reporting process identified. **Data quality:** could be optimised **System costs:** Development 275 000 Euro, annual costs about 235 000 Euro **Flexibility:** adaptable and transferrable. **Usefulness:** 89%: alarms stimulated activities, 84%: better at detecting febrile episodes than traditional surveillance. 83% of data analysis stakeholder missed a standardised response protocol. 100% agreed on adequate detection of outbreaks **Acceptability:** 48% feel time invested is not proportional to benefit, 24% believed not easy to use. **Stability:** 68% replied that the system was not available when needed, main barrier in data entry.	**Conclusions of study author:** - Specific areas of acceptability to enter data could be significantly improved - The high sensitivity and low specificity of syndromic surveillance is characteristic. This lack of specificity may lead to costly false alarms. - Sensitivity of the system means that if utilised correctly by s stakeholders, it is unlikely to miss any disease epidemic where the primary symptom is fever. - The timeliness and sensitivity were major strong points. - Logging of patients into the system took too much time
12. Flamand C et al. (2011) French Guiana January 2006–December 2010	In 2006 laboratory, sentinel, hospital and health centre-based surveillance was implemented, to improve early detection of outbreaks and to allow a better provision of information.	37 812 clinical cases and 10 724 confirmed cases analysed to validate the performances of the system.	a)30 voluntary general practitioners (35% of total GP's) b)Surveillance from Emergency Departments (EDs) of the three hospitals c)17 health centres weekly report number of cases by satellite.	1 Sporadic transmission 2 Dengue fever clusters 3 Pre-alert (exceeding threshold for 2 weeks) 4 Epidemic (exceeding threshold for 2 more weeks) 5 End of epidemic (below threshold) 6 Increase of positivity rates of biological analysis and re-emergence of a serotype is used to confirm the entry in the next stage.	- Three major outbreaks were detected - During these outbreaks, 80 signals were triggered for confirmed cases and 64 for clinical cases, all these outbreaks were confirmed - average duration of the epidemics varied between 38 and 41 weeks. - reinforcement vector control measures proportionate to the severity and magnitude of the epidemiological situation.	**Conclusions of study author** - Validity of the surveillance system and its performance to monitor dengue patterns, to detect outbreaks and to provide real-time information - Great variety of data sources constitutes a very sound basis for the analysis and interpretation - Time-series methodology and taking into account data characteristics such as secular trends, seasonality and abrupt changes should be considered in future. - Outbreak prediction in future will consist in the use of other data sources for surveillance such as environmental factors (i.e. climatic, meteorological, plant cover and land use) so as to help monitor and predict the spatial and temporal distribution of the virus.
13. Hoen Anne G (2012) (Dez. 2009–March 2011) The Americas	Investigated real-time electronic sources for monitoring spread of dengue into new regions.	Modelled outbreak probability density representing a risk map of recent DENV spread into areas of previously unknown dengue endemicity according to the 2010 Yellow Book by collecting outbreak data from HealthMap	We used receiver-operating characteristic analysis with cross-validation to set a threshold dengue report density that best identifies new dengue-endemic areas	Known dengue-endemic areas were defined as dengue risk areas identified by the US Centers for Disease Control and Prevention. Health Information for International Travel (commonly referred to as the Yellow Book), 2010 and 2012 editions	Of the 19 new dengue-endemic areas reported in the 2012 Yellow Book, this threshold identified 14 (74%) as being at elevated risk of endemicity, according to the dengue outbreak probability density estimated by our model. Of the 41 areas that remained unidentified as dengue-endemic areas in the 2012 Yellow Book, our model classified 35 (85%) as having reduced risk of endemicity. When compared with the Yellow Book, our model incorrectly classified 6 areas as at elevated risk	**Conclusions of study author:** Electronic event-based surveillance systems such as HealthMap and others are frequently used by public health authorities, travellers, physicians and patients, to gain a real-time understanding of global outbreak activity. Used in combination with traditional case reporting, HealthMap and other electronic surveillance systems have proven value for enhancing the timeliness of outbreak discovery and information dissemination (11). However, these information sources may also provide added value for monitoring ongoing spread.
14. Randrianasolo Laurence (2010) Madagascar 01.04.2007–31.12.2008	A sentinel syndromic-based surveillance system was set up in March 2007. The aim was to allow the rapid detection of an epidemic and to identify circulating arboviruses.	Challenges and steps involved in developing a sentinel surveillance system are described.	Uses health service-based indicators and mostly focuses on fever syndromes. Four sentinel primary health centres with high population densities were also implemented with arbovirus surveillance. Sentinel general practitioners (SGP) report weekly, using forms addressed within 24 h to the management team.	Fever (axillary temperature of more than 37.5 °C). Three illnesses in relation with fever were selected for surveillance: malaria, influenza-like illness, arbovirus (fever without respiratory symptom and at least two other symptoms: headache, arthralgia, myalgia-like backache, skin rash, retro-orbital pain, haemorrhagic manifestations).	In 2008, the sentinel surveillance system included 13 health centres. - Of the 218 849 visits to SGPs, 12.2% were related to fever syndromes. Of these 26 669 fever cases, 12.3% were related to dengue-like fever - 89% of cases have been reported within 24 h - Ten cases of fever clusters occurred; they were not detected by traditional surveillance system. Laboratory investigation confirmed the clinical signals. The sentinel surveillance system confirmed five outbreaks: two via an increase in the dengue-like syndrome ratio, one of chikungunya virus circulation; two Influenza A (H1N1 seasonal); one malaria	**Conclusions of study author:** - A sentinel real-time-like surveillance system may be the key to the detection of disease outbreaks. - Cost of data transmission was minimal, but maintenance requirements of the system need to be better quantified - The time required to conduct investigations might negate the advantage of timely data acquisition - This system cannot replace traditional surveillance - Epidemiological baselines for each centre need to be determined, to help develop better statistical methods and sensible alarm thresholds, which can then be extended to more sentinel centres.
*B. Surveillance for trend monitoring of dengue disease*						
15. Standish Katherine et al. (2010) Nicaragua, Managua 2004–2008	Laboratory-confirmed dengue cases identified through a Dengue Cohort Study (PDCS) were compared to those reported from other health facilities to the National Epidemiologic Surveillance (NES) programme.	To address the difference in dengue case capture rates between a paediatric dengue cohort study (PDCS) and the Ministry of Health dengue surveillance programme (‘expansion factor’) calculated.	The study captured possible dengue cases through ‘enhanced’ passive surveillance by study physicians and nurses at the HCSFV and periodic home visits for follow-up and monitoring. Inapparent DENV infections were identified through serological testing of paired annual blood draws from healthy subjects.	WHO criteria for suspected dengue, as well as undifferentiated fever. A dengue case was considered laboratory-confirmed when (i) DENV was isolated, (ii) DENV RNA was demonstrated by RT-PCR, (iii) seroconversion was observed (iv) a fourfold increase in antibody titre in paired sera.	- PDCS identified 14 to 28 (average 21.3) times more dengue cases each year per 100 000 persons than were reported to the NES. - Incidence of dengue ranging from 343 to 1759 cases per 100 000 persons in the cohort study, as compared to 21 to 77 cases per 100 000 persons across all Managua's health centres. - The ratio of inapparent to symptomatic DENV infection in PDCS participants also varied year to year from 16 in 2004–2005 and 2006–2007, to 5 and 3 in 2005–2006 and 2007–2008, respectively.	**Conclusions of study author:** - This was not a classic capture-recapture study, but rather an ecological study comparing incidence rates in a cohort to national surveillance rates in the surrounding urban areas. - The HCSFV district, which borders Lake Managua, may have higher dengue rates than other health centres, as there was more reported dengue among the non-study population of the HCSFV. - However, another plausible explanation is that these higher numbers are due to the impact of the PDCS study protocol and increased awareness. - The expansion factors falls in the same range as the only published expansion factors for ambulatory dengue. Meltzer et al. calculated that 10 and 27 times more DENV cases occur in Puerto Rico.
16. Wichmann Ole (2011) Cambodia Thailand	Estimation of the true burden if disease by calculating a multiplication factor.	To utilise laboratory-confirmed incidence of symptomatic DENV infection both in inpatients and outpatients identified in prospective cohort studies to estimate dengue under-recognition.	Cohort studies were conducted among children aged 15 years. Age group-specific multiplication factors (MFs) were computed. In Thailand, 14 627 person-years of prospective cohort data were obtained in two provinces and 14 493 person-years from one province in Cambodia.	Thailand: paired samples were obtained from all students with a history of fever within the previous 7 days or an oral temperature of >38 °C. Cambodia: Children (i.e. >38 °C, acute or in the previous 7 days) for 2 days (in 2006) or 1 day (in 2007), paired serum samples were collected	- Average annual incidence of laboratory-confirmed dengue was 23/1000 and 25/1000 in Thailand and 41/1000 in Cambodia. - Calculated MFs in these provinces varied by age group and year (range 0.4–29). In Thailand, a median 229 886 (range 210 612–331 236) dengue cases occurred annually during 2003–2007 and a median 111 178 (range 80 452–357 135) cases occurred in Cambodia in children <15 years of age. - Average under-recognition of total and inpatient dengue cases was 8.7- and 2.6-fold in Thailand, and 9.1- and 1.4-fold in Cambodia, respectively.	**Conclusion of study author:** - These data indicate that although dengue is regularly reported in many countries, national surveillance data significantly under-recognise the true burden of disease. - The major finding of our analysis was that dengue incidence was under-recognised by more than 8-times in Thailand and more than 9-times in Cambodia. - We conclude that the national surveillance systems in Thailand and Cambodia were efficient in capturing inpatient dengue case with only 2.6- and 1.4-fold under-detection, respectively. - However, the surveillance system in Thailand largely under-recognises the burden of dengue outpatients and the system in Cambodia does not allow reporting of outpatients at all.
17. Vong S et al. (2012) Cambodia Province Kampong Cham 2006–2008	Passive population-based surveillance system with active sentinel component versus a community-based active fever cohort.	Two-sample, capture–recapture study in the largest province in Cambodia to determine disease under-recognition to the National Dengue Surveillance System (NDSS).	Capture: Community-based active surveillance for acute febrile illness was conducted in 0- to 19-year-olds. Recapture: The NDSS is based on reporting of hospitalised, clinically diagnosed dengue cases aged **≤**15 years, reported passively from referral hospitals and at sentinel hospitals.	True dengue = febrile illness DENV-positive by serology or molecular testing. Dengue cases for the purposes of NDSS reporting were identified on a clinical basis using the 1997 WHO case definition.	- Of 14 354 individuals under active surveillance (22 498 person-seasons), the annual incidence ranged from 13. 4 to 57.8/1000 person-seasons. - During the same period, NDSS incidence rates ranged from 1.1/1000 to 5.7/1000, which was 3.9- to 29.0-fold lower than found in the capture–recapture study. - In hospitalised cases, the rate of under-recognition was 1.1- to 2.4-fold.	**Conclusions of study author:** - We conservatively estimated that there was a fourfold to 30-fold degree of dengue under-recognition and underreporting to NDSS - Under-detection levels changed from fourfold to 22-fold during the 2006 and 2008 non-epidemic years, and 29-fold during the 2007 large-scale epidemic year. -Under-recognition and reporting for hospitalised cases of dengue were much lower and generally more stable from year to year. However, during the large epidemic in 2007, underreporting was twofold higher than in other years. - Our findings show that NDSS appeared to accurately capture hospitalised cases over time.
18. Vong S et al. (2010) 32 villages and 10 urban areas of Cambodia during 2006–2008 during dengue seasons	Community-based active dengue fever surveillance among the 0- to 19-year age group.	To make a robust estimate of the actual incidence of symptomatic dengue virus (DENV) infection in children and adolescents living in rural and urban areas	Conducted by mothers trained to use digital thermometers and village teams (VT) from each respective village and five investigation teams (IT). VTs made weekly home visits to identify persons with fever or history of fever (axillary temperature of 37.5 °C).	A dengue case is defined as a febrile person positive for anti-DENV IgM in the convalescent-phase serum.	Over the three years, 6121 fever episodes were identified with 736 laboratory-confirmed dengue virus (DENV) infections for incidences of 13.4–57.8/1000 person-seasons.	**Conclusions of study author** - This active surveillance found a higher disease incidence than reported to the national surveillance system, particularly in preschool children and that disease incidence was high in both rural and urban areas. - It also confirmed the previously observed focal nature of dengue virus transmission.
*C. Trend monitoring and outbreak detection*						
19. Mark E. Beatty et al. (2010) 22 countries	Multiple approaches as in 22 countries practised.	Experts attended meetings to discuss dengue surveillance. Literature and reports on surveillance programmes were reviewed, and expert opinions shared.	- 12/22 (55%) of countries represented confirmed reported cases with laboratory testing. - Nearly every country includes suspected dengue cases. - Cambodia report children less than 15 years of age. - Singapore and Brazil monitor vector indices not so Puerto Rico. - All countries are dependent on paper forms for case reporting before any additional investigation or action.	Not applicable	- Surveillance and laboratory methods varied making comparisons difficult. - In Kolkata, special mapping has been used to target control activities. - In Singapore and Brazil, ministries using intranet-based data entry - Time required to receive a result of a sample is too long and so useless for the treating physician. - Data are rarely used locally missing the opportunity for a local response. - Under-detection and under-reporting of dengue cases were significant and often due to the design of the surveillance system. - Virological surveillance is under-utilised or completely lacking. - Effective links between the various stakeholders exists; many of these are dependent on personal contacts.	**Conclusions of study author** - Every dengue-endemic country should make reporting of dengue cases to the government mandatory. - Electronic reporting systems should be developed and used. - At minimum dengue surveillance data should include incidence, hospitalisation rates, deaths by age group. - Additional studies should be completed to check the sensitivity of the system. - Laboratories should share expertise and data. - Tests that identify dengue virus should be used in patients with fever for four days or less and antibody tests should be used after day 4 to diagnose dengue; - Early detection and prediction of dengue outbreaks should be goals for national surveillance systems.
20. Rekol Huy et al. (2010) Cambodia	Currently, national surveillance comprises passive and active data collection and reporting on hospitalised children aged 0–15 years.	This report summarises surveillance data on dengue collected in Cambodia since 1980. In addition, the impact of a 7-year vector control programme on the incidence of the disease was also evaluated.	NDCP gathered data reported passively from referral hospitals and collected actively at sentinel sites on weekly basis. Data were entered centrally into a computerised database.	Since 2002, clinical case definition of dengue fever and its complications have been based on World Health Organization (WHO) definitions and adapted for health centres and referral hospitals.	The alert system predicted the 2007 epidemic, the weekly incidence was consistently above the alert threshold of two standard deviations above the mean in early 2007; the response to the outbreak came too late. - No association between routine interventions and disease incidence was observed. - 57.0% in 2002 to 89.1% in 2008 of reported data came from sentinel sites. - Between 2000 and 2008, paired serum samples were collected from an annual mean of 715 patients, comprising 5.2% of dengue cases reported. Overall, 87.8% of samples were seropositive for dengue, and there was little variation across sentinel sites.	**Conclusions of study author:** The use of surveillance has several limitations: - Weaknesses in the design of the surveillance covered patients hospitalised at major public and non-profit-making paediatric hospitals and paediatric wards only. - Difficulty in classifying disease severity using standard WHO definitions - The size of the patient samples used in virological surveillance was small. - Patients were not selected randomly but because there was a high level of suspicion of having dengue - Dengue was frequently over-diagnosed during epidemics and under-diagnosed during the intervening periods. - Despite these limitations, our observation that dengue activity patterns for different ages and genders have remained consistent over time indicates that the surveillance data are reliable.
21. Ramos Mary (2008) Puerto Rico Patillas Municipality June 2005–May 2006	A laboratory-based, enhanced dengue surveillance system (EDSS) was developed and implemented at the health centre in the municipality of Patillas.	To provide a more accurate estimate of the incidence of symptomatic dengue and describe the clinical outcomes of dengue infection using data representative of this community.	Two full-time CDC staff members work at the health centre in Patillas to encourage HCPs to complete dengue case investigation forms and submit serum samples. CDC on-site staff verifies the accuracy and completeness of reporting and provide systematic feedback	World Health Organization (WHO) criteria to classify cases and applied a simplified case definition for severe dengue illness.	- A total of 1393 cases of suspected dengue were reported to the EDSS 156 (11.2%) were laboratory-positive, 422 (30.3%) were laboratory-negative, and 815 (58.5%) were laboratory-indeterminate. - 7.7 laboratory-positive dengue cases per 1000 population detected by EDSS were nearly three times higher than rates reported under the passive surveillance system during the two most recent epidemics in 1994 and 1998 (1.3 cases per 1000 in 1994 and 2.8 in 1998).	**Conclusions of study author:** Enhanced surveillance is useful for detecting population-based incidence of symptomatic infections - This surveillance does not detect asymptomatic infections or symptomatic infections among those who did not seek medical care. - Incidence of laboratory-positive dengue infection was high, particularly among adolescents and young adults. Although few cases met the WHO criteria for DHF, 10 times as many had at least one reported severe clinical manifestation, indicating that simplified case definition could be useful in clinic-based surveillance.
22. Schwartz Eli et al. (2008) Ill-returned travellers seen at GeoSentinel sites from Oct 1997–Feb 2006	GeoSentinel sites are specialised travel/tropical medicine clinics on 6 continents and 33 surveillance sites.	Seasonality and annual trends for dengue cases among 522 returned travellers are reported. Analysis over time was based on proportionate morbidity.	To be eligible for inclusion in the GeoSentinel database, patients must have crossed an international border and be seeking medical advice at a GeoSentinel clinic for a presumed travel-related illness.	Laboratory-diagnosed dengue in a resident of a non-dengue-endemic area who has travelled to a dengue-endemic area in the 14 days before symptom onset.	Among ill-returned travellers, 24 920 met the criteria for analysis. 522 (2.1%) had a diagnosis of travel-related dengue fever. - The increases in 1998 and 2002 were found entirely in travellers to South-East Asia; for 2003, in travellers to South Central Asia; and for 2005, in travellers to South Central Asia and Indonesia. These increases correspond to known epidemic years within local populations for those regions. - The major epidemic peak in sentinel travellers preceded the epidemic pattern in the local population during 1998 and 2002, as reflected in Thai reports to the World Health Organization.	**Conclusions of study author:** In April 2002, GeoSentinel alerted the international community of the increase in travel-related dengue from Thailand online. Data reported later confirmed the observation. The increase in dengue cases in returned travellers from South Central Asia in 2003 was also evident before official surveillance data were available. - A 2001 outbreak in Thailand apparently did not affect travellers, as it was not associated with a peak in reports to GeoSentinel. - Nevertheless, travellers may be sentinels able to rapidly inform the international community about the onset of epidemics in disease-endemic areas.
23. Domingo Christina (2011) European Travellers 2002–2008	Molecular surveillance in returning travellers.	To demonstrate the role of travellers as an additional source of epidemiological information complementary to countries data.	Samples were collected by virology research laboratories of the European Network or travel clinics, members of the European Network (TropNetEurop). Seven national reference laboratories participated	Suspected dengue case was defined as a patient with travel history in the previous 15 days to a dengue-endemic area, who presented fever plus two specified symptoms. Confirmation was carried out by molecular and serological diagnosis.	- 186 DENV strains (12 distinct genotypes) were detected in acute dengue-infected European travellers (82 DENV-1, 39 DENV-2, 48 DENV-3 and 17 DENV-4) - 10 new African strains are described. The detection of three different DENV serotypes in travellers returning from Cameroon pointed to a hyperendemic situation in the country in the absence of reported dengue haemorrhagic outbreaks. - The identification of the emergence of different serotypes and genotypes, the appearance of new clades correlating with outbreaks, and the identification of a dengue-4 genotype not previously reported have been achieved.	**Conclusions of study author:** - Returning travellers provided data even from areas with scarce DENV epidemiological information like African countries, - The increase in DENV correlating with observations in the respective countries (e.g. in Cuba, Ecuador, etc) - One of the main achievements was the detection of DENV-3 genotype I in Ecuador, confirming the recent detection of this genotype in the Americas. - We would like to remark that travellers constitute just a random sample and do not substitute the more comprehensive national surveys that would address the circulation of this genotype more accurately.
24. Runge-Ranzinger (2011) Cambodia Thailand	*Thailand*: *Passive population-wide reporting system* *Cambodia:* Passive population-wide reporting system of hospitalised paediatric cases and active sentinel sites.	Qualitative study based on key informant interviews and secondary data analysis. Aim: To study the practical application of dengue disease surveillance, analyse programme response and their interlinkages.	*Thailand*: passive integrated reporting of clinical confirmed cases mainly public (indoor) sector. Serological surveillance at 6 sites, 3% cases laboratory confirmed. Cambodia: passive, paper-based integrated reporting of suspected hospitalised paediatric dengue patients, public sector exclusively. Virological surveillance at 5 sentinel sites implemented, data analysed, 10% cases laboratory confirmed.	*Thailand:* *Hospital: Fever+ pos. TT (Tourniquet Test) = suspected; + Leucopenia (<5000) =confirmed. 2 confirmed DHF per village in 28 days = outbreak.* *Median of past 5 years-20%=outbreak.* *Cambodia:<15 years, 38–40 °C fever, mucocutaneous haemorrhagic signs or positive TT.* Outbreak: mean of cases (over past 3 years) plus 2 SD (Standard Deviations)	**Sensitivity**: *Thailand*/Cambodia: Underreporting: *shown by studies to be 40% in a hospital/*1:3 hospitalised and 1:5–6 for total paediatric cases are strong underestimations. **Sensitivity of alert**: *20% lowered threshold and additional local definition increased sensitivity/*observation of excess reporting in low transmission season could potentially be used in addition. **False alerts**: non-experienced applying the thresholds above. **Timeliness:** *4 weeks*/6–7 weeks **Usefulness:** both for national planning yes, for outbreak detection too late **Preparedness:** Both no contingency plans or alert algorithms available. Lack of linkage from data to response. **Response***: Mainly case based, response teams implemented/*no effect between routine interventions and transmission could be demonstrated, lack of resources.	**Conclusions of study author:** Low sensitivity due to (i) low user rates, (ii) clinical assessment only, (iii) reporting limited to public sector, certain age groups or inpatients only, (iv) limited acceptability at all levels and (v) an insensitive case classification. Timelines could be improved by (i) reporting of suspected cases, (ii) avoid double reporting and compiling, (iii) the use of prompt to fill forms, (iv) data analysis at all levels, including district, (v) data entry already at district level, electronic reporting. Other recommendations: a) Establish a common understanding of all stakeholders on the surveillance purpose and objectives, b) Ensure a close linkage of analysed surveillance data to evidence-based response, bedded in proper contingency planning, c) Increase additional active/sentinel/syndromic components based on a clear rational, d) Further research on appropriate thresholds/alert indicators or a risk assessment tools is needed.
25. Carolina Fracalossi Rediguieri 2009 Bolivia Brasil (Goiás State)	*Bolivia*: *Passive population-wide reporting system.* Brazil: Passive population-wide reporting system and active sentinel sites. ^*^ the mean incidence of each epidemiological week is calculated by taking into account the incidence in the two previous weeks and in the two weeks after that epidemiological week	Qualitative study based on key informant interviews and secondary data analysis. Aim: To study the practical application of dengue disease surveillance, analyse programme response and their interlinkages.	*Bolivia*: passive integrated reporting of suspect and laboratory-confirmed cases mainly public sector. Active search of severe cases (after an index case). 10% cases laboratory confirmed. Brazil: passive integrated reporting of suspect and laboratory-confirmed cases mainly public sector. Border sentinels (passive), active search for virus circulation 63 PHC (passive), active search for severe cases, after index case. 10% cases laboratory confirmed.	*Bolivia*: Number of cases 1.24 times above the median of the past 5 years. Endemic area + fever + anorexia and nausea or skin eruptions or headaches or leucopenia or positive TT= suspect case; serology or PCR = confirmed Brazil: Mobile mean^*^ of the past 5 years + 2SD; incidence above 300 cases/ 100 000 inhabitants. Endemic area + acute fever (up to 7 days) + 2 or specific symptoms.	**Sensitivity**: *Bolivia*/Brazil: *Underreporting in both countries*. **Sensitivity of alert**: *not good for outbreak detection or prediction /*high for outbreak detection. **False alerts**: not experienced applying the thresholds above. **Timeliness:** *45 days*/3 weeks. **Usefulness:** both for national planning yes, for outbreak detection too late **Preparedness:** Both contingency plans available, but no alert algorithms available. **Response**: *Mass interventions, such as fumigation and social mobilisation through campaigns/*Response targets the vector (larvae control, ULV), urban cleaning, social mobilisation, case detection and management.	**Conclusions of study author:** Low sensitivity of case detection due to: (i) the existence of asymptomatic dengue or undifferentiated febrile illness, (ii) patient's non-care seeking behaviour, (iii) poor access to health facility, (iv) low specificity of case classification, (v) average acceptability of the system, (vi) reporting limited to public sector, certain age groups or inpatients only. Timelines could be improved by (i) avoiding double reporting and compiling, (ii) analysing data at all levels, (iii) electronic reporting. Recommendations: a) A simple and standardised case definition b) The establishment of criteria for selecting patients for virus circulation to increase its representativeness; c) The establishment of active virological and serological surveillance d) Feedback of data reported should be improved e) Development of accurate triggers that will allow the early response to epidemics; f) Research on development of indicators for outbreak prediction; g) Development of algorithm for outbreak declaration
26. Novarti I 2010 Indonesia West Java in Java Island and Lampung in Sumatera Island.	Passive population-based reporting system. Active surveillance system in some sentinel primary health care	Semi-structured interviews with key informants and secondary data analysis Aim: to explore the existing surveillance system and analyse programme response	Passive reporting of dengue cases. No data available on how many per cents of the reported cases were laboratory confirmed. Virological surveillance only for research purpose.	Hospital: Clinical examination (fever, rash/torniquet test) + thrombocytopenia+ Haemagglutination test positive for dengue or NS1 Outbreak: Increasing cases by twofolds or more compare with same period of last year or a new case at a place where there were no dengue cases previously.	**Sensitivity**: cases reported from hospital only in 30% reached the district health office **Sensitivity for alert**: excess reporting in interepidemic session usually be used as an early alert. **False alert**: non-experienced applying the threshold before. **Timeliness**: 3–4 weeks **Usefulness**: for national planning yes, for outbreak detection too late **Preparedness**: No contingency plans or alert algorithms available. **Response**: No linkage between routine control and transmissions. **Data quality**: incomplete data are the main problem **Representativeness**: Dengue patients seeking treatment at the health facilities estimated only 30%.	**Conclusions of study author** - Low sensitivity is due to (i) low user rate, (ii) clinical examination only, (iii) not all health facilities report the cases to public health authorities. - Timeliness is regarded too late to predict outbreaks - Timeliness and data quality could be improved by (i) simple data form, (ii) data analysis including lowest possible level, (iii) also private health centres should report all suspected cases (iv) integrated data reporting Other recommendations: a) Case classification is too insensitive. b) Study on health-seeking behaviour and treatment response as well as perceptions of health staff regarding dengue cases and dengue surveillance c) Common understanding on purpose and objectives of the surveillance systems by all stakeholders d) Data flow and reporting lines more consistent e) Further research on appropriate thresholds/alert indicators is needed
27. Aishath Aroona Abdulla 2011 outbreak Maldives	Objective of the surveillance system not clearly defined. Passive population-wide, integrated, manual reporting system. So far not clearly mandatory.	Evaluation based on 7 interviews and secondary data analysis. To identify room for improvement after the 2011 outbreak.	Daily reporting of clinical suspected/confirmed dengue patients via fax, E-mail, telephone according to Communicable Disease Notification Form (varies from hospital to hospital) in paediatric and internal health facilities.	Old WHO case definition. Laboratory rarely available. No outbreak definition applied.	**Sensitivity:** Reporting rate of selected hospital in Mai 2011: 54%, lower for outpatients, mild and adult cases. **Sensitivity of alert**: Cases were above the previous mean since December 2010. Shooting up than around week 25 in 2011. Outbreak declaration then beginning of June 2011 **Timeliness** of case notification: up to 4 days **Usefulness:** For monitoring yes, for outbreak detection threshold/trigger was missing-late alert. **Representativeness:** Variable due to atolls commitment **Acceptability:** Low at all levels - Late dissemination of data - Little data analysis capacity	**Conclusions of study author:** - Agree on specific objectives for surveillance - Change to the new WHO case classification (2011) - Revise reporting form, include instructions and harmonise them. - Reporting needs to be mandatory - Implement laboratory component and eventually surveillance - Manual reporting should be electronically wherever possible - Regular training especially on case reporting and data analysis and outbreak detection - Earlier data dissemination - Regular evaluation of the system
*D. Low and non-endemic countries monitoring of imported cases, detection of autochthonous transmission*						
28. Gobbi Federico (2012) Italy Veneto Region	In 2010, a special surveillance for West Nile virus (WNV), dengue virus (DENV), and chikungunya virus (CHIKV) was initiated in the Veneto Region of north-eastern Italy.	The (pilot study) surveillance had 2 main objectives. To (i) increase the detection rate of imported CHIKV and DENV in travellers and identify autochthonous cases, (ii) detect autochthonous cases of WNF	Possible cases detected by general physicians and emergency department physicians had to be referred within 24 h to the closest Unit of Infectious or Tropical Diseases. Serum samples were sent to the regional reference laboratory (Padua, Italy) for confirmation.	Suspected: Fever >38 °C during the past 7 days in a traveller who had returned within the previous 15 days from an endemic country, absence of leucocytosis and other obvious causes of fever. Probale =NS1 rapid test positive. Confirmed: PCR, Serology or NT positive	Of 79 possible cases, we detected 14 cases of DENV infection and 1 case of CHIKV infection among travellers with fever. No cases were severe. No autochthonous case of fever caused by DENV has been documented in Italy.	**Conclusions of study author:** The proportion of virus-positive patients was strikingly high: ≈20% of persons tested who had imported fever were positive for DENV or CHIKV, as were 10% of persons with locally acquired fevers for WNV. Compared with the 2 previous years, the special surveillance enabled detection of substantially more cases, showing that you only find what you are looking for. The success of this pilot phase prompted regional authorities to propose a 3-year plan as part of the integrated surveillance of arboviral diseases, along with animal and entomologic surveillance
29. Gjenero Margan et al. (2011) Croatia	Enhanced surveillance and survey after alert by IHR. Routine reporting not stated. Presumably not mandatory, passive.	Case study: The information about a returning German traveller received from RKI (**30.09.11**) on the first autochthonous case of dengue fever was sent to the World Health Organization (WHO) via the International Health Regulations (IHR) information network.	A circulatory letter informing all services and hospital infectology clinics in the country to consider the possibility of dengue fever in clinically compatible cases including those with no history of travelling. 14 blood samples from neighbours and 112 from anonymous patents were examined.	Not stated	- In the following weeks, a number of clinically suspect cases were reported, and serum samples were sent to the CNIPH, but tested negative for dengue virus. - **22 October 2010**, a possible case of dengue fever was reported in a resident of the same village where the German patient had stayed, then confirmed by paired sera with increase in IgM and IgG. - From the 14 samples, nine were positive for IgG and 7 had positive or borderline results for IgM - From the 112 samples, 6 had positive IgG (5.4%) and five positive or borderline IgM.	**Conclusions of study author:** - After France, Croatia is the second country in Europe in which autochthonous transmission after 1927/28 in Greece. - Although until recently dengue fever was not a notifiable disease in Croatia, it is unlikely services would have missed the occurrence of a confirmed case of imported dengue fever. - Each cluster of infectious diseases is reported using the national communicable diseases early warning system: During summer 2010, there were no such reports from Pelješac. Only four of the DENV-positive villagers contacted health services for febrile illness in August and September and were not recognised as an outbreak.
30. Ruche G la et al. (2010) France	Passive routine reporting Enhanced surveillance (May to November) since 2006 Laboratory surveillance system (most sensitive)	Case study describing the first two autochthonous cases in France and public health measures subsequently implemented.	Laboratory surveillance detected 350–400 imported dengue cases /year (2006 –2009). In the same period, enhanced surveillance reported 33 imported dengue cases. Between 1.5 - 17.9.2010, 120 imported cases of dengue have been reported by the enhanced surveillance system (11-fold increase)	Not stated	- Case one: Nice (23.08.2010): detected by enhanced surveillance - Case two: Nice (11.09.2010): 70 m from the first case, hospitalised for fever of unknown origin. - Level 2 of the national contingency plan was activated after the first case: - Level 3 was activated after the cluster was identified	**Conclusions of study author:** - The event was not unexpected and a specific preparedness plan timely developed. - The high vector density in Nice and the increase in the number of imported cases (due to the epidemic in French West Indies) are two major factors - The reactive surveillance in addition to the routine enhanced surveillance is likely to identify new symptomatic cases in the area. - This event shows the advantage of preparedness in order to implement rapid and proportionate measures of surveillance and response.
31. Hyo-Soon Yoo (2009) Korea 2001–2006	Passive population-based surveillance system.	The aim was to identify the timeliness of Korean National Notifiable Disease Surveillance System (NNDSS).	The NDDS is an electronic (since 2000) reporting system covering 50 diseases (in 4 categories) since 2008 organised at three levels: local, provincial and central. Reporting is expected within 1 (group 1 + 4) or 7 (group 2 + 3) days.	Not stated	- The median time from disease onset to notification to KCDC ranged between 6 and 20 days. - The median time from onset to registration at the local level ranged between 2 and 15 days. - Most time lags arose from a delay in diagnosis, especially for typhoid fever (T1, 10 days), dengue fever (T1, 10 days) and shigellosis (T1, 5 days). Dengue fever that represents Group IV showed the longest delay in TC and TR, primarily due to delays in both steps of diagnosis (T1) and doctor's report (T2).	**Conclusions of study author:** Time from disease onset to diagnosis generally contributed most to the delay in reporting. - Electronic reporting systems can be an important means to enhance timeliness. - One of the main reasons for the variation in reporting among different diseases is the clinical characteristics of the diseases such as mode of onset and severity. - The necessity for time-consuming laboratory tests for diagnosis may also delay reporting
32. Mei-Mei Kuan (2010) Taiwan 1998–2007	In 2003, active surveillance for dengue was integrated into the airport fever screening programme to reduce the importation of DENV strains.	This study aimed to examine the epidemiological trends and the impact of imported cases and airport fever screening on community transmission. The impact of implementing airport fever screening was evaluated.	During 1998–2002, airport screening for DENV was implemented in the form of a questionnaire filled out by all passengers. Following thermal scanning by non-contact infrared thermometers to detect those whose body temperature was >37.58 °C, blood samples were tested by molecular and/or serological diagnosis.	Imported cases: cases reported by local clinics or airport fever screening with a travel history in the previous 2 weeks, whereas the indigenous cases were defined as cases reported by local clinics without any travel history.	- A total of 10 351 dengue cases, including (7.1% imported) between 1998 and 2007. - The majority of indigenous dengue cases (98.5%) were significantly clustered in southern Taiwan; 62.9% - Peak season was Sept to Nov. - Airport fever screening was successful in identifying 45% of imported dengue cases with fever. - No statistical impact on community transmission comparing presence and absence of airport fever screening. - Dengue cases appeared to be positively associated with population density (RID–PD = 0.4–0.6) and population number (RID–PN = 0.5–0.7) in the epidemic years of 2002, 2006 and 2007.	**Conclusions of study author:** - 55.0% (298/542) of imported cases were temporary, non-febrile, that is, latent cases, undetected by airport fever screening. - 50–90% of dengue infection cases are asymptomatic, and therefore, transmission of DENV into Taiwan via incoming travellers may be inevitable. - The efficacy of screening symptomatic passengers passing through Taiwan airports by NCIT was found to have a PPV = 30.5–62.6% when fever prevalence among passengers was <1%. - The effect on mitigating community transmission in dengue epidemics was not significantly different between pre-2003 border control methods and post-2003 methods (fever screening).
33. Chien-Chou Lin et al. (2009) Taiwan 2002–2007	hospital-based reporting system and hospital syndrome reporting system for viral haemorrhagic fever. Active surveillance systems (individual self-suspected reports, expanded epidemiological contact surveys, school and community screening systems, airport fever screening).	Unlike sero-epidemiological studies, the data presented in this study were derived from routine diagnosis and analysed anonymously.	Report completed within 6–24 h then online available for the local health bureau and hospital. Once the case is confirmed, sheet is completed by the local health bureau or hospital. Staff will visit and interview the index case. Blood samples will be drawn from contacts within a radius of 50 metres, those who may have had contact or had fever.	A confirmed dengue case is defined as (i) positive for dengue virus isolation; or (ii) positive for dengue virus genome by RT-PCR; or (iii) positive for dengue virus-specific IgM and IgG in a single serum sample, or (iv) fourfold increase of IgG antibody in paired samples.	First indigenous index case usually occurs May or June, imported from South-East Asia. - Eventually, the outbreak then spreads out gradually and peaks around October and ending in the winter. - 3 to ˜6 dengue virus strains imported and locally transmitted each year. Only one strain of serotype has dominated in each year. - This pattern has been repeated yearly -42 150 blood samples were drawn for serological analysis, of which 1.1% (464/42 150) were found to be dengue virus infected. All 464 persons did not feel sick enough to go to the hospital for medical treatment. - ratio of symptomatic to asymptomatic cases is 1.78 (64%/36%)	**Conclusions of study author:** - Dengue in Taiwan is an adult infectious disease; elderly have high morbidity and mortality rates. - Secondary infection increases the disease severity, but not mortality, dengue-infected adults are more symptomatic. - The transmission cycle in Taiwan is unique, beginning with importation in the summer and ending in the winter. - Most (64%) of the dengue-infected persons showed clinical symptoms after DENV-1 /DENV-3 infection. - Severe disease can be caused by secondary infection with DENV-2 or primary infection with DENV-1 or DENV-3.
34. Huang Jyh-Hsiung et al. (2007) 2005 Taiwan	(i) passive (hospital-based reporting) and (ii) active (fever screening at airports, self-reporting, screening for contacts of confirmed cases, patients with fever of unknown origin, school-based reporting) surveillance systems.	Presentation of the results of a laboratory-based dengue surveillance and phylogenetic study in Taiwan for 2005. Human samples used were derived from confirmed dengue cases submitted to the Taiwan CDC in 2005.	Dengue is a category 2 reportable infectious disease in Taiwan. Suspected cases must be reported within 24 h using the old WHO classification scheme. Surveillance systems are established by central and local health departments in Taiwan.	Laboratory diagnosis: Infection with DENV was defined as a febrile illness associated with the detection of DENV-specific IgM and IgG antibodies, isolation of DENV or detection of DENV RNA by reverse-transcription–polymerase chain reaction (RT-PCR).	- A total of 104 laboratory-confirmed imported dengue cases were identified in Taiwan during 2005, 46 (44.2%) cases were identified by fever screening at airports. - Similar to the findings of our previous study, Indonesia, Vietnam, the Philippines and Thailand were the most frequent importing countries - A total of 202 laboratory-confirmed indigenous dengue cases were recorded in Taiwan during 2005. 12 DENV-1, others DENV-3 (two strains) and DENV-2.	**Conclusions of study author:** - Laboratory-based dengue surveillance system to identify febrile patients at the airports by an infrared thermal scanner. Most (44 of 46) of the confirmed cases identified by airport fever screening were in the viraemic stages. These 34 cases were identified on days 1–3 after onset of illness. - In contrast, the imported cases reported from passive (hospital) surveillance systems were evenly distributed 1–20 days after the onset of illness. - Phylogenetic analyses suggested that the three epidemic strains, DENV-3, genotype I, DENV-3, genotype II and DENV-2, American/Asian Genotype) which cocirculated in southern Taiwan in 2005, were recently imported from the Philippines and Vietnam, respectively.
35. Shu Pei-Yun et al. (2009) 2003–2007 Taiwan	(i) passive (hospital-based reporting) and (ii) active (fever screening at airports, self-reporting, screening for contacts of confirmed cases, patients with fever of unknown origin, school-based reporting) surveillance systems.	Presentation of the results of a laboratory-based dengue surveillance and phylogenetic study in Taiwan for 2003–2007. Human samples used were derived from confirmed dengue cases submitted to the Taiwan CDC in 2003–2007.	Dengue is a category 2 reportable infectious disease in Taiwan. Suspected cases must be reported within 24 h using the old WHO classification scheme. Surveillance systems are established by central and local health departments in Taiwan.	- Imported dengue case =infected patient travelling abroad > 2 weeks before the onset of illness. - Indigenous case= when overseas travel is not indicated - DENV infection=febrile illness with the detection of DENV-specific IgM and IgG antibodies, the isolation of DENV by RT-PCR.	- A total of 542 imported dengue cases were identified in Taiwan during 2003–2007. Among them, 17 (28.8%), 57 (62.6%), 46 (44.2%), 48 (44.0%) and 75 (41.9%) cases were identified by fever screening at airports from a total of 59, 91, 104, 109 and 179 imported cases for 2003, 2004, 2005, 2006 and 2007, respectively. - With the increasing trend of imported dengue cases, we also witnessed larger dengue outbreaks in Taiwan resulting in 965 and 2000 indigenous dengue cases in 2006 and 2007, respectively	**Conclusions of study author:** - > 95% of the imported patients detected by fever screening at airports are in their viraemic stage. - Among these imported cases, 74% cases were identified on days 1–3 after onset of illness. - In contrast, the imported cases reported from passive (hospital) surveillance systems were evenly distributed 1–20 days after the onset of illness. - the distribution of the countries of origin accurately reflected the frequency of air travel between Taiwan and these nations, as well as dengue outbreaks during the same period in the country of origin. - Geographic distribution of strains and genotypes of DENV-3 isolated from South-East Asian countries remain unchanged during 2003–2007. - However, the movement and new establishment of DENV-1, DENV-2 and DENV-4 strains were observed in certain areas of Asia.
36. Mei Mei Kuan and Feng-Yee Chang (2012) (2007–2010) Taiwan	The active surveillance includes fever screening at the airport (since 2003) within others. The passive surveillance refers to the hospital-based reporting system for the notification of either imported or domestic dengue	This study is intended to assess the performance of the airport screening procedures for dengue infection	Travellers with a thermal NCIT-detected temperature of higher than 37.5 °C were detained at the entry gate, rechecked by quarantine officers with a survey and reassessed using an ear thermometer. Travellers with a temperature above 38 °C were defined as confirmed fever cases.	Confirmed dengue case = positive RNA, antigen or antibody by laboratory diagnoses. Domestic dengue case= confirmed case not travelled in the two weeks prior to the onset. Imported =confirmed case travelled to endemic countries two weeks prior to illness.	44.9% (95% CI: 35.73–54.13%) of the confirmed imported dengue cases with an apparent symptom (febrile) were detected via the airport fever screening programme, with an estimated positive predictive value of 2.36% and a negative predictive value > 99.99%. Additionally, the fluctuating patterns in the cumulative numbers of the imported dengue cases with 1–2 months lead time (t) was in parallel with that of the domestic dengue cases based on a consecutive 4-year surveillance.	**Conclusions**. The screening programme could assist in the rapid triage for self-quarantine of some symptomatic dengue cases that were in the viraemic stage at the borders and contribute to active sentinel surveillance; however, the blocking of viral transmission to susceptible populations (neighbours or family) from all of the viraemic travellers, including those with or without symptoms, is critical to prevent dengue epidemics. Therefore, the reinforcement of mosquito bite prevention and household vector control in dengue-endemic or dengue-competent hot spots during an epidemic season is essential and highly recommended.

As many studies were descriptive or ‘ecological studies’ and therefore could not be ranked according to the ‘hierarchy of study designs’ (York 2001), the National Health and Medical Research Council (NHMRC 2000) evidence hierarchy (Merlin *et al.* 2009) was used to group the studies according to study design. Only studies at evidence ‘level IV or level III-2 and III-3’ were included. Studies were grouped according to study types: models, time-series, case studies, ecological studies, evaluations, expert consensus, descriptive studies, prospective and retrospective cohorts.

No studies were excluded in the analysis for quality reasons if the eligibility criteria were met, and the limitations and possible biases in such studies are reported in the results section. The analysis grouped studies into four categories based on the purpose of the surveillance approach under investigation: (A) outbreak prediction/detection; (B) trend monitoring; (C) both outbreak prediction/detection and trend monitoring; and (D) low/non-endemic countries.

## Results

**Figure 1:**
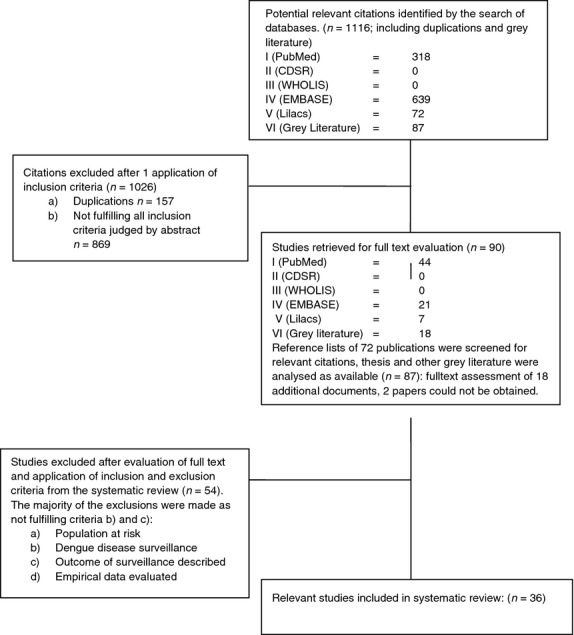
Flow chart of articles included and excluded.

A total of 1116 studies, including duplicates, were identified during the electronic search as potentially relevant to the research question. After screening of titles and abstracts, 90 studies remained eligible. Full assessment of the text eliminated 54 further studies, leaving 36 studies included (Figure1). Data of the 36 studies were extracted to a table (Table1), also assigning a unique identifier number for each study.

When grouped according to purpose of the surveillance system studied and compared with the 2008 review, an increase in research interest in early outbreak detection was apparent, particularly in endemic countries: (A) outbreak prediction or detection (14 studies/previously 5); (B) monitoring dengue trends (4 studies/previously 6); (C) outbreak prediction and trend monitoring (9 studies/previously 7); and (D) non-endemic countries (9 studies/previously 6). Within each of these categories, key components essential for improving surveillance in endemic and non-endemic countries were identified. The detailed findings are summarised and presented in Table1.

### Surveillance systems for outbreak detection and/or prediction (Groups A and C)

Most of these studies were from highly endemic settings and were intended to predict or detect outbreaks at an early stage.

#### Using electronic event-/search query-based surveillance for early detection of increased dengue activity

Two studies investigated the value of data quantifying the numbers of internet searches seeking dengue information in a number of countries (Bolivia, Brazil, India, Indonesia and Singapore; studies 1 and 2), by comparing with epidemiological data from the surveillance system using time-series analysis. The curve of the search queries over time was similar to the epidemic curve constructed from surveillance data, underlining the usefulness of this new and relatively simple approach. Study 13 used a real-time electronic approach based on Health Map in order to enhance timeliness and outbreak detection and to provide an added value for monitoring the ongoing spread of dengue.

#### Using the appearance of a new dengue serotype/genotype as an alert signal for dengue outbreaks

Six studies investigated serotype changes as a dengue outbreak signal (studies 3–8) using virus surveillance information, analysing laboratory data (genotyped or sequenced data) or hospital data (severity of cases) and examining how these correlated with the number of reported cases or dengue incidence. Five of these studies (3–7) also analysed whether a serotype shift or a clade replacement was positively associated with a subsequent epidemic. The sixth study investigated the correlation between population-wide serotype-specific data and an increase in cases (study 8) and showed that outbreaks occurred following the introduction of new serotypes in specific islands.

Examining these studies in detail, retrospective studies in Singapore (studies 3, 5) found that a serotype switch from DENV-2 to DENV-1 in 2004/2005 was associated with the 2005 epidemic. However, according to Schreiber (study 6), viral genome sequencing would not have been sufficient to predict this outbreak. A switch from DENV-1 back to DENV-2 in early 2007 was used as a warning sign and led to response actions that were believed to have reduced the impact of an outbreak 6 months later. A clade replacement within DENV-2 was also considered a contributing factor to the 2007 Singapore outbreak (study 3) and another outbreak at the end of 2010 (study 4). Similarly, three surveys in Surabaya (Indonesia) investigated prospectively the correlation of DENV type and disease incidence. Here, an increase in case numbers in 2010 was attributed to a genotype shift in DENV-1 from genotype IV to I between April and September 2009 (study 7). Retrospective analysis of serotype-specific surveillance data in the Pacific region (study 8) demonstrated that the rapid replacement of DENV-1 by DENV-4 in the region was associated with dengue outbreaks in 2008 and 2009 in Kiribati, New Caledonia, Samoa, Tonga and other islands.

#### Using syndromic surveillance to create alert signals for dengue outbreaks

Five studies investigated the value of syndromic surveillance for early outbreak detection. These included a comparison of community-based fever surveillance with surveillance of school absenteeism in Peru (study 9) and two studies in French Guiana (studies 10 and 12) that described the advantages of reporting dengue cases using a syndromic case definition compared with routine reporting. These two French Guiana studies and another in Madagascar (study 14) used sentinel sites and reported higher sensitivity and outbreak early warning capacity compared with the routine reporting systems (which were based on laboratory surveillance and passive case reporting). Studies 10 and 12 highlighted the need for maintaining the traditional surveillance and considering the increased potential for false alerts in syndromic surveillance systems.

The prospective study in Peru indicated that community door-to-door fever surveillance had higher sensitivity than school absenteeism records as an indicator for dengue (study 9); the community-based fever cohort captured twice as many cases as the school-based approach.

In French Guiana (study 10), the syndromic clinical surveillance in a military population and the routine laboratory reporting systems were found to be complementary: the syndromic approach detected an outbreak 3–4 weeks earlier and was six times more sensitive than laboratory-based surveillance, but the specificity was lower in the former. Further analysis (study 11) using CDC criteria (CDC 2001) showed that the ideal reporting time was often not achieved due to barriers at data entry and that an increased risk of false alerts needed to be considered. However, all respondents perceived that this system detected outbreaks adequately and subsequent countrywide introduction of sentinel-based syndromic reporting in French Guiana identified 80 signals for confirmed cases and 64 for clinical cases and predicted three major epidemics (study 12). In Madagascar, a sentinel-based syndromic surveillance system for six diseases was evaluated: it detected ten outbreaks, five were confirmed and two of which were dengue (study 14).

#### Use of other sentinel site-based approaches to increase capacity for outbreak detection

Three studies analysed sentinels sites for early outbreak warning, either in the form of sentinel-based reporting and virus surveillance (Cambodia, study 20) or for non-endemic countries (studies 22, 23, see group D below). One study described an enhanced routine surveillance system in Puerto Rico by motivating public health staff, which resulted in an increase in reported dengue incidence three times above the incidence during the two most recent epidemics in 1994 and 1998 (study 21). In Cambodia, passive surveillance plus sentinel site surveillance including virus surveillance increased the sensitivity of detecting outbreaks (defined as numbers of cases exceeding two standard deviations [SD] above the mean) although the response was delayed, mainly due to inadequate financial management (study 24).

In Europe, ten new strains of dengue viruses were detected in travellers returning from Africa, and increased observation of dengue in travellers by surveillance networks (TropNetEurop) was correlated with outbreaks documented in national data (study 23).

### Surveillance for describing endemic/epidemic trends (Group B and C)

These surveillance systems under investigation were mostly population-based and passive. Some included additional sentinel sites or virus surveillance but they were used only to monitor viral trends and were not applied to early warning.

Four cohort-based studies calculated the level of underreporting, either using capture–recapture approaches comparing two independent surveillance systems or by comparing cohort-based data with the national routine reporting. The expansion factor indicating the level of underreporting was calculated to be:

- 14–28 times in Nicaragua for a paediatric cohort (study 15)
- 8.7 times in Thailand (2.6 times for hospitalised cases) (study 16)
- 9.1 times in Cambodia (1.4 for hospitalised cases) (study 16, 18)
- 3.9–29 times in Cambodia following a capture–recapture analysis (study 17)
- 1.1–2.4 times in Cambodia following a capture–recapture analysis of hospitalised cases (study 17)

The results demonstrated remarkably high levels of underreporting in the surveillance systems, particularly for non-hospitalised cases. It was a common experience that a large proportion of the affected population was not captured by passive routine reporting (e.g. non-users of health services, users of private/traditional sectors or certain age groups (e.g. adults in Cambodia).

Four evaluations of routine dengue surveillance systems (studies 24–27) in 6 countries (Brazil, Bolivia, Cambodia, Indonesia, Maldives and Thailand) were conducted using a similar protocol for evaluations based on CDC Guidelines (CDC 2001). Both trend monitoring and outbreak detection were evaluated. All evaluations found that a clear understanding of the objectives of the surveillance system by all stakeholders was crucial. The routine reporting systems – some of them with laboratory support – were perceived to be useful for trend monitoring and national planning but, as they did not apply appropriate thresholds/alert signals or include additional surveillance components, they had little capacity for early outbreak detection. In particular, reporting timeliness was perceived to be low, ranging from a few days for notification in the Maldives (study 27) to six to seven weeks until data analysis in Cambodia (study 24). Moreover, the responses were delayed, as shown in the Maldives, where no threshold for taking action was implemented, and in Cambodia, where lack of sufficient financial management and other constraints undermined any response to the alert signal of ‘increased transmission (above two SD) in low transmission season’. In Thailand, where the system relied exclusively on clinically confirmed cases, respondents felt that outbreak responses were delayed because decision-makers did not trust the data and feared false alerts (study 24). All evaluations reported that timeliness could have been increased by electronic reporting or simplified reporting forms and that data analysis should have been performed at the lowest possible level (e.g. every district, once per week), given that sufficient capacity was available.

### Dengue surveillance in low/non-endemic countries (Group D)

The value or effectiveness of primarily laboratory-supported active dengue surveillance systems in non-endemic settings was described in several studies from Asia and Europe. Timeliness of the system and laboratory support were reported to be crucial elements.

Three European studies described the recent detection of dengue in France (study 30) and in Croatia (study 29) and imported dengue cases in Italy (study 28). In Croatia, the notification of returning travellers led to the detection of autochthonous cases, while survey-based investigations revealed additional cases (29).

An evaluation of routine reporting in Korea (study 31) reported a 2- to 15-day delay from disease onset to reporting, which was shortened when electronic reporting components were introduced.

Four studies from Taiwan (studies 32–36) demonstrated the effectiveness of linking routine reporting with strong laboratory support and active and syndromic reporting elements in monitoring epidemiological, virological and clinical trends. Airport fever screening (studies 32 and 36) detected around 45% of imported dengue cases, but any impact this might have had on subsequent autochthonous transmission could not be determined.

## Discussion

### Key findings

A greater number of the studies included in the present study (19/36) were performed in Asia than in the Americas (8/36; previously 17/24), illustrating a shift in research attention to Asia from the Americas since the 2008 review, when 17 and 6 studies, respectively, of 24 were recorded. In the present study, four studies (4/36) had a global focus, one study was from Africa and three studies were from Europe, most likely reflecting the global spread of, and consequent interest in, dengue disease in these regions in recent years.

### Tools for trend monitoring (Group B and C), and as baseline for ‘excess reporting’ for outbreak detection

The surveillance systems deployed for this purpose were mainly population-based and passive. Some included additional sentinel sites or virus surveillance, but in those cases, the data were used only to monitor viral trends and were not applied to early warning. Four cohort-based prospective studies calculated an expansion factor with a range between 1.1 and 2.6 for inpatients in Cambodia and Thailand, respectively, and between 3.9 and 29 in Cambodian, Nicaraguan and Thai cohorts for non-hospitalised cases.

The results demonstrate remarkable levels of underreporting in the surveillance systems, particularly for non-hospitalised cases. It was a common experience that a large proportion of the affected population was not captured by passive routine reporting (e.g. non-users of health services, users of private/traditional sectors or certain age groups, e.g. adults in Cambodia). However, while less than satisfactory, this does not mean that such a system is entirely inadequate, because as long as it is accurately reflecting the disease trend, it may still be used effectively as a baseline for detecting excess reporting (e.g. more than 2xSD above the mean of the previous 5 years) and thus outbreak detection. In the context of a public health system, it is not clear how sensitive surveillance data need to be (i.e. what is an acceptable level of under-reporting) in order to fulfil the dual purposes of reflecting disease trends accurately and providing a baseline for outbreak early alert. The studies reviewed here indicated that underreporting to a limited extent can be tolerated in high endemic settings, as long as the data are geographically representative and, ideally, laboratory confirmed as dengue. The calculation of an expansion factor enables a more accurate value for the national burden of disease, which is important for targeting public health measures and advocacy.

In the earlier systematic review (Runge Ranzinger *et al.* 2008), the sensitivity of the DF/DHF/DSS case classification was considered to be too low (studies 20, 24–26), especially for DHF cases (Bandyopadhyay *et al.* 2006). With the new WHO dengue case classification, described in the WHO dengue guidelines (World Health Organization and the Special Programme for Research and Training in Tropical Diseases (TDR) 2009) and the Handbook on Clinical Management of Dengue (WHO 2012), this problem has been overcome, because the new WHO dengue case classification classifies according to disease severity, permitting more sensitive reporting of severe disease and allowing comparison of data across all regions (Barniol *et al.* 2010, Horstick *et al.* 2012, Horstick *et al.* 2014) as described in study 27.

Four evaluations of routine dengue surveillance systems (studies 24–27) in 6 countries (Brazil, Bolivia, Cambodia, Indonesia, Maldives and Thailand) were conducted using similar protocols for trend monitoring and outbreak detection, based on the CDC Guidelines (CDC 2001). However, all evaluations found that a clear understanding of the objectives of the surveillance system by all stakeholders was crucial. All routine reporting systems, with or without laboratory support, were perceived to be useful for trend monitoring and national planning. However, without the use of appropriate thresholds or alert signals or additional surveillance components to increase timeliness or sensitivity (e.g. as sentinel sites or syndromic surveillance components), they had little capacity for early outbreak detection. Improvements indicated by the evaluations were not exploited.

An appropriate alert signal with a defined threshold level (‘trigger’) for initiation of a response is crucial for any system. None of the reviewed studies investigated the specific threshold for excess reporting within a routine surveillance system. However, analysis of the included articles suggested that in general, an excess of reported cases (pattern recognition technique; Buehler *et al.* 2004) – identified through a population-based routine surveillance system – has potential for dengue outbreak prediction. Studies that evaluate sensitivity, specificity and positive predictive values of such a threshold are likely to be particularly valuable.

Throughout the studies, reporting time was slow, and without any threshold, responses were delayed while poor financial management and lack of trust in the data by decision-makers hindered further the delivery of adequate and timely response measures. Despite that, all evaluations reported that timeliness could have been increased by electronic reporting or the use of simplified reporting forms and that data analysis should have been performed at the lowest possible level (e.g. once per week in every district) if sufficient capacity was available.

In summary, the country evaluations consistently highlighted that immediate improvement is possible using a number of options, many of which are already available and easily implementable: (i) simplified data forms/data entry protocols/electronic-based reporting, (ii) clearly defined and easily understood system objectives, (iii) appropriate and regular/frequent data analysis at the lowest possible level (iv) and regular data feedback from top to bottom levels. As evidence becomes available, two additional components will be required to complete the model: (i) clearly defined and locally appropriate triggers for an outbreak response (no studies were found exploring the optimal sensitivity and specificity of such thresholds) and (ii) implementation of evidence-based response strategies.

### Alert signals^1^ (triggers/indicators/thresholds) for epidemic response (Group A, C and D)

Predicting outbreaks through the introduction or shift of a dengue sero-/genotype: six studies (studies 3–8) investigated serotype changes as a dengue outbreak signal demonstrated a positive correlation with the number of reported cases or dengue incidence, although the lag times could extend up to 6 months. However, viral genome sequencing alone would, according to Schreiber (study 6), not have been sufficient to predict an outbreak.

But these events are highly site-specific and are influenced by herd immunity, population size, co-circulation of additional dengue viruses and potentially numerous other factors. Moreover, only those countries with reliable serotype-/genotype-specific surveillance would be able to monitor changes in any patterns. Genotypic shifts were used as an early warning signal in Singapore prior to the 2007 epidemic and initiated an early response (study 3). Taking into consideration the possibility that publication bias (i.e. that only positive results are likely to be published) would have excluded additional studies where serotype shifts were not associated with subsequent outbreaks and that numerous potential confounding factors would have been possible in all studies, and it is not yet possible to draw any firm conclusions on the value of this as a measure in surveillance. Nonetheless, the sensitivity, specificity and positive predictive value of this parameter merit evaluation in prospective and comparative studies.

Predicting or detecting dengue outbreaks by syndromic surveillance data: Five studies investigated the value of syndromic surveillance for early outbreak detection. These included a comparison of community-based fever surveillance with school absenteeism in Peru (study 9), and two studies in French Guiana (studies 10 and 12) describing the advantages of reporting dengue cases using a syndromic case definition as compared to routine reporting. The prospective study in Peru indicated that community door-to-door fever surveillance had higher sensitivity than school absenteeism records. In French Guiana (study 10), the syndromic approach detected an outbreak 3–4 weeks earlier and was six times more sensitive than laboratory-based surveillance, but specificity was lower. However, in another study in French Guiana (11), the ideal reporting time of 60 min for a real-time syndromic surveillance approach was often not achieved due to barriers at data entry, while a risk of false alerts was expected, given the high sensitivity of the system. In Madagascar (14) and French Guiana (12), syndromic sentinel-based surveillance built on clinical syndromic case definitions showed promising results, increasing the sensitivity of dengue case detection in comparison with routine reporting and allowing the early detection of epidemic events.

Two studies investigated the value of data quantifying internet searches for dengue information carried out in a number of countries (Bolivia, Brazil, India, Indonesia and Singapore; studies 1 and 2). The curve of the search queries over time was similar to the epidemic curve constructed from surveillance data underlining the usefulness of this new and remarkably simple approach. Study 13 used a real-time electronic event-based approach based on Health Map to enhance timeliness, outbreak discovery and provide an added value for monitoring the ongoing spread of dengue.

A number of studies that were included in the earlier 2008 review also dealt with this topic; in summary, the following syndromic surveillance-based indicators were identified:

- Proportion of virologically confirmed cases (study 3, Rigau-Pérez & Clark 2005)
- Malaria negative rate in fever patients in a malaria endemic areas (Carme *et al.* 2003, Talarmin *et al.* 2000)
- Fever alerts (Pirard *et al.* 1997; Kourı *et al*. 1998)
- Clinical syndromic case definitions (study 10, 11, 12 and 14)
- School absenteeism (study 9)
- Google search queries or event-based surveillance (Study 1, 2 and 13)

Fever alert for the purpose of outbreak detection was not found to be useful in Cuba and Bolivia (Pirard *et al.* 1997; Kourı *et al*. 1998). None of the studies included in this update analysed syndromic surveillance based on laboratory parameters or the proportion of virologically confirmed cases. One study from Singapore (study 3) mentioned that during the 2007/2008 epidemic, the proportion of DENV-positive samples detected by PCR rose from 57.9% in January 2007 to 91.0% in July 2007 at the peak of transmission. A similar trend has been shown in Puerto Rico previously (Rigau-Pérez & Clark 2005).

In summary, detection of increases in proportions of positive tested samples and quantification of electronic search queries are both promising approaches to dengue outbreak detection. They are inexpensive and offer near real-time data and their value for operational use should be considered and investigated. Syndromic surveillance based on a clinical case definition remains a complementary tool to national routine reporting.

## Limitations

The main limitation of this review was its restriction to English, German and Spanish. However, as the bulk of literature accessible on electronic databases today is indexed in English by title and abstract, and no additional articles in other languages were found during the extensive search, the impact of a language bias is likely to be limited. While publication bias is a potential concern, by screening carefully the reference lists of assessed articles and grey literature, the bias has been reduced.

A ‘research hot spot’ in Singapore and Taiwan was identified: these two countries accounted for 10 of the total of 36 studies, potentially introducing some level of bias in the overall assessment of the published literature. Potential for bias also may have occurred with respect to the evidence demonstrating an association between newly introduced dengue serotypes and subsequent outbreaks (see below), because no studies reporting the absence of any association (i.e. new serotypes not followed by an increase in dengue; a phenomenon that is arguably, less likely to be published) were found.

Two key knowledge gaps were identified: none of the studies investigated whether the thresholds currently in use for triggering an outbreak response were at an appropriate level of sensitivity or geographical scale, and none indicated how outbreaks were distinguished from standard or ‘expected’ seasonal changes in transmission. Further research in this area remains of the highest priority and is strongly recommended.

## Conclusions

Following the systematic review of the evidence of the value or potential of various tools or approaches in for dengue outbreak prediction or trend monitoring, the following conclusions can be drawn:

- Passive surveillance remains the backbone of disease monitoring, also providing the baseline for outbreak alert. All opportunities for improvement should be exploited to ensure that disease trends are accurately reflected. While underreporting could be tolerated to a certain extent, further research will be required to determine how much.
- The usefulness of the new dengue case classification for epidemiological use should be evaluated, as it is currently underway for its clinical use.
- Country evaluations of dengue surveillance systems should be conducted and published following CDC criteria.
- More research is necessary to identify appropriate thresholds of excess reporting that can be used to trigger an outbreak response; such studies must take into account both the geographical scale as well as the level of sensitivity.
- Appropriate additional alert signals need to be identified and tested and integrated risk assessment tools need to be developed.
- Additional well-designed and well-implemented enhancement tools (such as active surveillance components, laboratory support or motivation strategies) would strengthen surveillance.
- Shifts in dengue serotypes or genotype have considerable potential in dengue surveillance, and the value of these data merits evaluation in prospective and comparative studies. It is crucial that both negative and positive results be published to overcome publication bias in favour of positive associations.
- Syndromic surveillance approaches have potential as useful complementary tools offering increased timeliness and sensitivity but with an increased risk of false alerts. Further studies investigating laboratory parameters (e.g. the proportion of confirmed-to-requested laboratory tests) are also merited. Internet searches or electronic event-based surveillance strategies also show promise, although their operational usefulness remains to be demonstrated.
- Further research on evidence-based response strategies and cost-effectiveness is still needed.

## Note

Alert signals for an unexpected increase in dengue case numbers (an ‘outbreak’) could include signals from within the dengue time series itself, or signals from external indicators associated with changes in dengue transmission. Other indicators (additional to the increase in cases) could be considered as indicators for a dengue outbreak. (Note: ‘Alert signals’ – also called alarm signals – are indicators that, at a defined threshold, are intended to ‘trigger’ a response).
